# Femtosecond laser induced low propagation loss waveguides in a lead-germanate glass for efficient lasing in near to mid-IR

**DOI:** 10.1038/s41598-021-90249-9

**Published:** 2021-05-24

**Authors:** Mamoona Khalid, George Y. Chen, Heike Ebendorff-Heidepreim, David G. Lancaster

**Affiliations:** 1 Laser Physics and Photonics Devices Laboratory (LPPDL), University of South Australia, Mawson Lakes, SA 5095 Australia; 2 Institute for Photonics and Advanced Sensing and School of Physical Sciences, University of Adelaide, Adelaide, SA 5000 Australia

**Keywords:** Materials for optics, Optics and photonics, Lasers, LEDs and light sources, Optical materials and structures

## Abstract

To support the growing landscape of near to mid-IR laser applications we demonstrate a range of low propagation loss femtosecond laser (FSL) written waveguides (WGs) that have achieved guided-mode laser operation in a rare earth (RE) doped lead-germanate glass. The WGs are fabricated in both the athermal and thermal FSL writing regimes using three different pulse repetition frequencies (PRF): 100 kHz (athermal); 1 MHz; and 5 MHz (thermal). The lasing capability of Yb^3+^ doped lead-germanate waveguides is verified in the near-IR. The refractive index contrast (∆n) for 100 kHz WGs is ~ 1 × 10^–4^, while for 5 MHz, ∆n increases to ~ 5 × 10^–4^. The WGs in the thermal regime are less effected by self-focusing and are larger in dimensions with reduced propagation losses. For the 1 MHz repetition rate thermal writing regime we report a low propagation loss WG (0.2 dB/cm) and demonstrate laser operation with slope efficiencies of up to ~ 28%.

## Introduction

Direct inscription of waveguides (WGs) into laser gain materials using a femtosecond laser (FSL) is a fast fabrication technique that allows complex photonic functionalities to be integrated into a single device^1^. These WGs can realize lasers that are ideal sources for photonic networks because of their small cavity size, moderate average power (~ 100 s of mW), and long energy storage lifetimes^2^. FSL allows for writing embedded low propagation loss symmetrical structures within the transparent materials (glasses) by tight focusing of the ultrashort laser pulses beneath the surface of the material.

Germanate glasses are fascinating hosts to achieve new laser operating regimes as this glass provides a good balance of properties required for efficient laser operation in the short to mid infrared region^3–7^. This includes longer wavelength transmission of germanates into the infrared region, competitive thermal, chemical and mechanical strength, medium phonon energy (~ 800 cm^−1^), and high refractive index compared to the widely researched silicates^8^ and fluorides^9^ (where very low propagation loss WGs have already been reported). Despite the above-mentioned properties, FSL-based WG writing in germanates for laser development has only been minimally investigated. Germanate is a good candidate for further research to identify the suitable FSL parameters that can introduce low propagation loss guiding structures and high laser slope efficiencies in near to mid-IR regions.

The reported studies of FSL written WGs in germanate cover the low and high pulse repetition frequencies (PRF) ranging from 1 kHz to 1 MHz. Early work reported a 1 kHz FSL (80 fs pulses) single line WG inscribed in Er^3+^ doped lead-germanate glass GeO_2_–PbO–Ga_2_O_3_^10^, however the measured propagation loss was high at 4.8 dB/cm. Operating at a higher PRF (500 kHz, 350 fs pulses) resulted in bright guiding structures in a fluorogermanate glass possessing a low propagation loss of ~ 0.7 dB/cm^11^. In other work, single line WGs inscribed in a widely investigated Barium Gallo-Germanate (BGG) glass resulted in propagation losses of as low as 0.5 ± 0.1 dB/cm for WGs inscribed using 250 kHz, 70 fs laser pulses^12^. Stress-induced WG writing (double line) has also been achieved in germanate by a low PRF FSL (4 kHz) in Er^3+^ doped GeO_2_–PbO–Ga_2_O_3_ resulting in 2 dB/cm of propagation losses^13^ i.e. the propagation losses reduced by a factor of 2 with double line writing compared to single line as in^10^. Similar stress induced (double line) WGs include a Nd^3+^ doped GeO_2_-PbO waveguide amplifier exhibiting a propagation loss of ~ 1.75 dB/cm; and in^14^ the authors reported an internal gain of ~ 4.6 dB/cm for double line WGs in an Er^3+^/Yb^3+^ co-doped germanate waveguide amplifier (10 kHz PRF). Overall, it is apparent that to achieve efficient germanate laser operation reductions in WG propagation losses in germanate glasses are required.

One of the challenges of FSL writing in germanate glass is its high 1st order (n) and 2nd order refractive index (i.e. nonlinear index, n_2_). The high linear refractive index of the germanate glass (1.8–2) is not matched with standard (n ~ 1.5) index matching liquid (e.g. oil) and oil immersive objective lenses which are designed to compensate spherical aberrations in the focal region. Furthermore, irradiating FSL pulses can exceed the self-focusing critical power limit of the glass which can occur even at low average laser power in conjunction with other FSL parameters^15^. This combination typically results in non-spherical and elongated structures. Currently there are no commercially available high-index matching oils and objective lenses; thus limiting the parameter space that can be explored.

In this paper, we investigate a range of FSL inscribed low propagation loss single line and double line induced WGs in a rare earth doped lead-germanate glass GeO_2_-PbO-Ga_2_O_3_-Na_2_O (hereafter referred to as GPGN glass) and verify their near-IR lasing capability. The selection of this composition of GPGN glass is based on the fact that its low loss fabrication has extensively been researched for mid-IR fibers^7,16,17^. Employing GPGN glass for this study extends its utilization to inscribe low loss waveguides for laser applications.

The aim of the study is to optimize the FSL parameters in such a way that low propagation loss guiding structures are induced in the GPGN glass. Yb^3+^ is doped in the glass to evaluate the lasing performance of the fabricated WGs. We believe that this study will provide a road map towards the fabrication of low loss WGs for efficient lasing in the mid-IR. The study is conducted for both the athermal (low PRF) and thermal (high PRF) regimes. The effect of nonlinear interaction of FSL with GPGN glass and the resulting modified index region in athermal (100 kHz) and thermal (1 MHz and 5 MHz) regimes of FSL are discussed, and the parameters to achieve lowest propagation loss in GPGN glass are presented. A high slope efficiency of ~ 28% is achieved for 1 MHz double line inscribed WG which is ~ 5 times higher than achieved with FSL parameters presented in our previous study^16^.

## Experimental methods

### Glass fabrication and waveguide writing

Yb^3+^ doped GPGN glass ingots are fabricated by a conventional melt quench technique^18^. The ion density of Yb^3+^ selected for small cavity WG laser operation is ~ 7 × 10^20^ ions/cm^3^. The 15 × 12 × 4 mm^3^ glass sample is cut to have parallel surfaces. The sample is polished to optical grade for WG writing, characterization, and laser demonstration.

**Figure 1:**
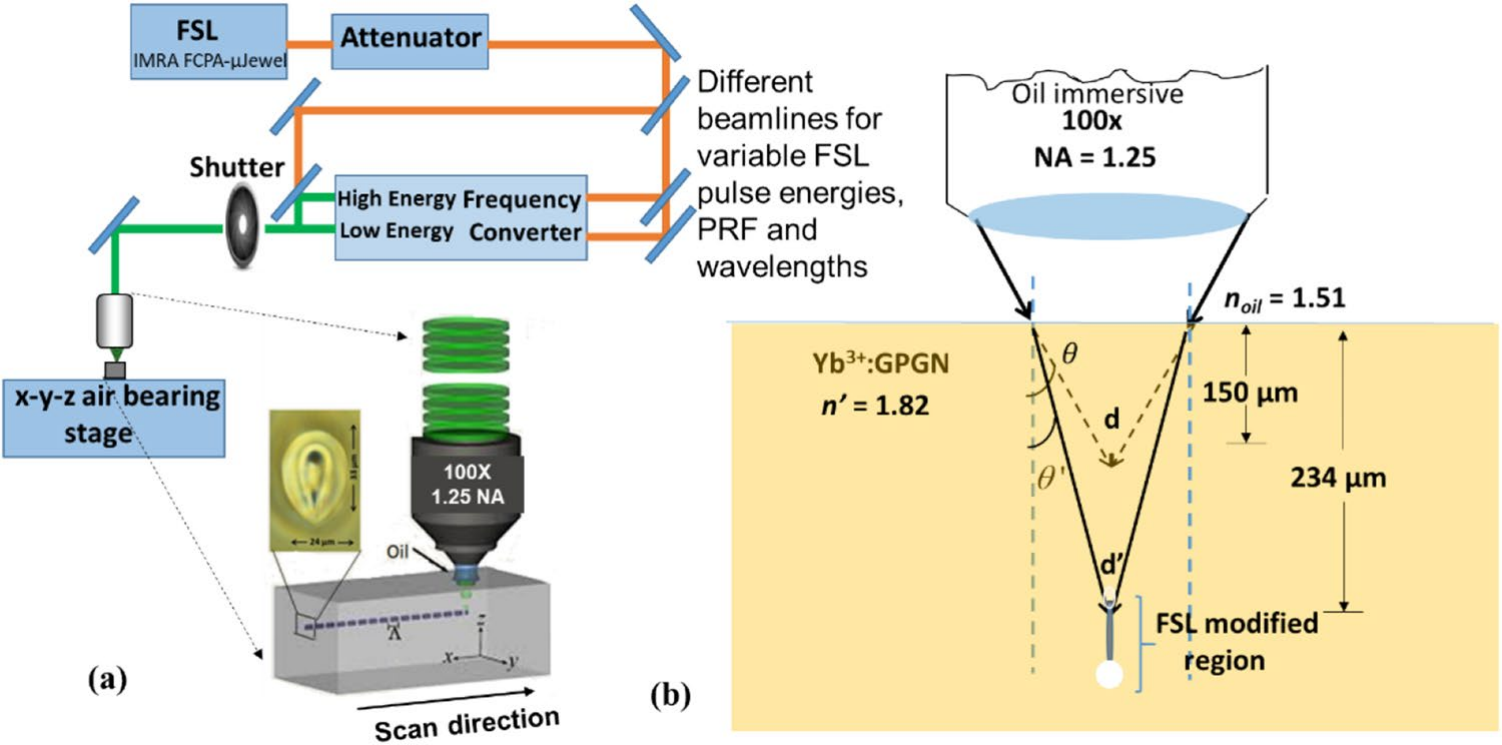
(**a**) FSL material processing setup for WG writing. The sample is moved transversely with respect to the FSL focus to inscribe tracks/lines of index modifications. The cross-section of the single line FSL modified region presented in the sample is the heat accumulated region (thermal writing regime). Reproduced with permission from ^20^. (**b**) Ray schematic showing the estimated change in writing depth [from d = 150 μm to d’ = 234 μm (FSL estimated focus)] through a low refractive index n = 1.51 index matching oil to a high refractive index sample (n’ = 1.82). θ and θ’ are the angle of refraction of the focusing beams (for actually set and estimated writing depths) with respect to incidence normal.

The parameters to achieve controlled and symmetric refractive index modifications in glasses depends on the careful tuning of the FSL parameters such as pulse energy, translation speed, PRF, and the laser spot size^19^. These parameters need to be balanced with glass properties (e.g. thermal conductivity) to produce modifications that are unique to each glass composition. For writing WGs in GPGN glass a ~ 250 fs pulse width Yb^3+^ fiber laser (IMRA FCPA-µJewel) operating at 1047 nm (P_avg._ = 2.4 W) and externally frequency doubled to λ = 524 nm is used. The WG writing setup is demonstrated in Fig. 1a.

The glass sample is translated in three dimensions using an air-bearing x–y–z translation stage. An initial translation speed of 0.5 mm/s is selected for these experiments based on the value around which a minimum propagation loss in BGG germanate glass was achieved (~ 0.5 dB/cm)^21,22^. Moving the sample transversely with respect to the focus of the FSL produces tracks (also known as lines) of respective index modifications at the focus point all the way through the sample (the dashed line in Fig. 1a represents single line writing at the FSL focus). Multiple parallel tracks (e.g. double lines, triple lines etc.) with a defined spacing between the lines are also inscribed with the aim to increase the cross-section of the resulting WGs. If assuming n = 1.5 (the design wavelength for the 100 ×, NA = 1.25, oil immersive microscope objective), the linearly polarized laser beam would be focused 150 µm beneath the surface of the sample with a predicted FSL spot radius of ω_0_ ~ 0.13 µm (${\upomega }_{0}=\uplambda /\mathrm{\pi NA}$) (Fig. 1).

Selected writing depth into this n = 1.82 glass is a balance between shallow writing depths resulting in thermally induced cracking, while deeper focusing increases the spherical aberration. An externally set writing depth of 150 μm is chosen which results in an estimated writing depth of ~ 234 μm (Fig. 1b) predicted using Snell’s law. After WG writing, the end faces of the sample are polished back by 1 mm each to reveal the cross-section of the WGs.

### Waveguide characterization

**Figure 2:**
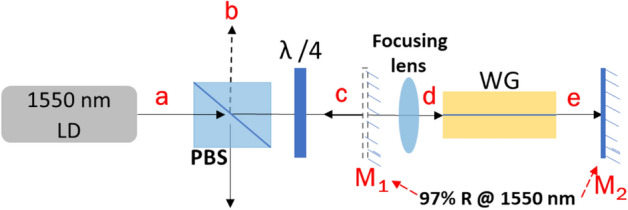
Experimental configuration to measure CL and TL (and thus PL) through a WG. A 1550 nm LD is used as the probe beam. λ/4 is the quarter waveplate, PBS is the polarized beam splitter. M_1_ and M_2_ are the 97% reflecting mirror @ 1550 nm, placed at point ‘**c**’ and ‘**e**’ without and with sample respectively. Probe beam loss in the forward beam path (‘**a**’ to ‘**e**’) is measured at point ‘**e**’ (details in supplement 1) and the beam loss in the reflecting path (from ‘**e**’ to ‘**b**’) is measured at point ‘**b**’.

WG characterization focuses on the investigation of (i) structural features of the modified region; and measurement of (ii) coupling loss (CL) into the WG; (iii) propagation loss (PL) through the WG determined from the measured transmission loss (TL); and (iv) numerical aperture (NA) of the WG. For the structural characterization, the cross-sectional details of the modified region are collected via rear illuminated brightfield microscopy. To characterize the WG losses (CL, TL etc.) a flexible probe beam technique is developed that can simultaneously measure CL and TL. The alignment error is estimated by taking 4 measurements of each WG. Figure 2 shows the complete setup employed for the loss measurements.

A single transverse mode, linearly polarized 1550 nm fiber coupled LD is used to probe the WGs. 1550 nm is chosen as the Yb^3+^ doped glass does not have ground state absorption at this wavelength. A polarized beam splitter cube (PBS-104) followed by a quarter waveplate (λ/4) is used to separate the probe beam going-to and traveling-back from the glass sample (Fig. 2). A red He–Ne laser counterpropagating to the probe light ensured the optical components are orthogonal to the direction of the probe beam. The probe beam is a ~ 0.8 mm diameter (D4σ) collimated beam focused into the WGs using a 10 mm focal-length lens to produce (D4σ) a ~ 16 µm beam spot.

With reference to Fig. 2, the total probe beam loss (L), in units of dB, in the optical path from ‘**a**’ to ‘**e**’ includes the following loss contributions:

(i) Optical loss (OL) through the PBS and the λ/4 waveplate.
(ii) Mirror M2 loss (ML) while retroreflecting the probe beam to ‘b’.
(iii) Coupling loss (CL) which arises due to the mode mismatch between the probe beam and the WG dimensions and WG NA. A reasonable mode-match is selected based on a f = 10 mm focusing lens.
(iv) Fresnel reflection loss (FL) due to the surface reflections at the air/glass/air interfaces of the glass sample.
(v) Transmission loss (TL) due to the scattering, impurities, and defects in the WG.

The probe beam is then retroreflected back to ‘**b**’ from ‘**e**’ to allow determination of TL*,* independent of CL (based on the assumption that the light that propagates in the waveguide to ‘**e**’ is already mode matched). The probe beam is retroreflected at ‘**e**’ via butting a R = 97% mirror to the end of the glass sample. The 3% leakage from the butted mirror has the advantage of allowing the launched mode to be monitored at ‘**e**’. A phosphor-coated CCD camera beam profiler (Ophir SP503U, factory calibrated to be linear at 1550 nm) is used to measure the beam intensity of the WG fundamental mode by selecting it using the built-in aperture of the beam profiler software (pixel values integrated over the circular aperture). CL and TL are calculated based on measured beam intensities at selected locations in the experimental layout using the derived loss equations given in detail in the supplement 1. The PL of a WG is then extracted from the measured TL using PL = TL/d, where d is the length of a WG in cm. The final set of equations through which TL and CL are calculated are given below

1 $${\text{TL (dB) = L}}_{{4}} - {\text{ L}}_{{3}} - {\text{ L}}_{{2}} - {\text{ L}}_{{1}}$$

2 $${\text{CL}}\left( {{\text{dB}}} \right){\text{ = 2L}}_{{3}} {\text{ + L}}_{{1}} - {\text{ L}}_{{4}}$$

where L_1_ = OL + ML, L_2_ = FL, L_3_ = CL + TL + FL, and L_4_ = 2TL + 2FL + CL + OL + ML.

The NA of a WG is estimated by the 1550 nm probe beam divergence angle (θ) out of the WG. Using this estimated NA of the WG, and the refractive index of GPGN glass, the refractive index modification is estimated using ${\Delta \text{ n = }}{{{\text{ (n}}_{{{\text{core}}}}^{{2}} - {\text{ n}}_{{{\text{cladding}}}}^{{2}} {)}} \mathord{\left/ {\vphantom {{{\text{ (n}}_{{{\text{core}}}}^{{2}} - {\text{ n}}_{{{\text{cladding}}}}^{{2}} {)}} {{\text{2n}}_{{{\text{core}}}}^{{2}} }}} \right. \kern-\nulldelimiterspace} {{\text{2n}}_{{{\text{core}}}}^{{2}} }}$^16^.

### Waveguide laser setup

**Figure 3:**
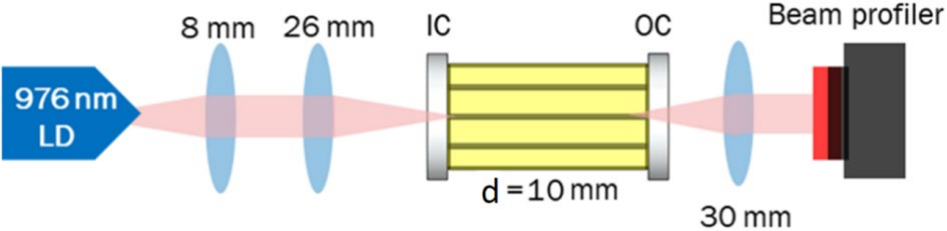
Laser setup to demonstrate the near-IR WG laser operation. The laser cavity consists of a HT @ 976 nm IC mirror and a 90% reflecting OC mirror @ ~ 1 μm. Cavity length is d = 10.5 mm.

Figure 3 shows the experimental configuration for demonstrating the WG laser. A 900 mW, 976 nm fiber coupled LD pump beam is launched into the laser cavity using a pair of achromatic lenses configured for mode matching to the targeted WGs. The WG laser cavity consists of a highly transmitting (HT) input coupler (IC) at 976 nm and a 90% reflecting output coupler (OC) @ ~ 1 µm butted to the end facet of a WG giving a laser cavity length of d = 10.5 mm. The diverging laser beam from the WG is collimated using a 30 mm aspheric lens to monitor the mode profile using the camera-based beam profiler (SP503U).

## Results and discussion

### Fresnel reflection-based loss (FL) and optical loss (OL) in Yb^3+^: GPGN glass

With reference to Fig. 2, OL from ‘**a**’ to ‘**e**’, then back to ‘**b**’, is measured under conditions where the focusing lens and glass sample is removed. OL is measured to be ~ 0.43 dB which includes 0.13 dB of M_2_ loss.

The GPGN glass sample is then inserted into the setup (without the focusing lens) to determine FL. The probe beam is passed through the GPGN bulk glass (i.e. the portion of the sample without WGs). The FL due to the air/glass/air interfaces of the sample is measured to be 0.73 ± 0.05 dB. The experimentally determined FL is in close approximation to the theoretical value of FL = 0.74 dB which is calculated from the refractive index of GPGN glass (n = 1.82) using FL = 10 × log ((n^2^ + 1)/2n). The value of n is taken from^4^.

### FSL interaction and self-focusing in GPGN glass

FSL-based WG writing involves tightly focused FSL pulses that produce extremely high peak powers P_FSL_ (P_FSL_ = E/τ_FSL_, E = FSL pulse energy and τ_FSL_ = FSL pulse duration) in the focal volume of a glass sample that eventually leads to permanent local modification of the refractive index^23^. Due to these peak powers at the focal volume, a non-linear multiphoton absorption takes place resulting in the release of an electron. Once a bounded electron is free through the initial ionization process, it then undergoes avalanche ionization where the free electron linearly absorbs the remaining laser pulse, gets excited and transfers its energy to the neighboring atom which then releases another electron once it has attained sufficient energy. The avalanche ionization process, therefore, results in exponential growth of free electrons, which generates a plasma and melts the localized volume of the sample. Due to the very short lifetime of free electrons (of the order of ps^23^), the electrons decay to the valence band almost immediately. This rapid cool down of the melted glass (fast quenching) thus freezes the structural modification and can lead to a change in the refractive index of the focal volume.

The high peak-power of the FSL pulses also induce nonlinear self-focusing into the material at the FSL exposed area. The self-focusing effect increases with increasing pulse energy until a critical self-focusing power (P_critical_) is reached, where it counters the diffraction and produces an elongated structure as the beam continues propagating deeper into the glass^24^. The critical self-focusing power is given by Eq. (3)

3 $${\text{P}}_{{{\text{critical}}}} = \frac{{3.77{\text{ }}\lambda ^{2} }}{{8\pi {\text{nn}}_{2} }}$$

where $\uplambda$ is the operating wavelength of the FSL, n and ${\mathrm{n}}_{2}$ are the linear and nonlinear refractive indices of the material, respectively. The self-focusing critical limit in germanate glass is reached at low pulse energies due to its high ${\mathrm{n}}_{2}$ value compared to fluoride and silica^15^. To estimate the P_critical_ in GPGN glass the nonlinear refractive index (${\mathrm{n}}_{2}$~ 56 × 10^–20^ m^2^ W^−1^) of a related lead-germanate glass (GPLN) from^25^ is considered. Applying n_2_ = 56 × 10^–20^ m^2^ W^−1^, n = 1.82, and λ = 524 nm in Eq. (3) gives ${\mathrm{P}}_{\mathrm{critical}}$ ~ 40 kW for lead-germanate. For comparison with silica where n = 1.45 and n_2_ = 2.4 × 10^–20^ m^2^ W^−1^^26^, P_critical_ is considerably higher ~ 1183 kW.

### Athermal regime (100 kHz repetition rate)

For WG writing in the athermal regime, the PRF of the FSL is set to 100 kHz. The time between the successive 100 kHz laser pulses τ_athermal_ is 10 μs, which we assume is longer than the thermal diffusion time, τ, of this glass. τ can roughly be estimated from the thermal diffusivity of the glass given by α (in m^2^/s) = κ/(ρ C_p_) where κ is the thermal conductivity, ρ is the glass density and C_p_ is the specific heat capacity. To evaluate α for GPGN glass we consider κ = 0.7 Wm^−1^ K^−1^ (for a lead-germanate glass in^27^), ρ_GPGN_ = 5.61 g/cm^3^^16^ and C_p_ = 500 J/(kg K) (for another germanate glass in^28^). The focusing conditions for the FSL used in this study (focusing radius ~ 0.2 μm for λ = 524 nm and NA = 1.25) leads to τ ~ 0.8 μs for GPGN glass which is comparable to the thermal diffusion time of glass mentioned in^29^ (τ ~ 1 μs, for λ = 800 nm and NA = 1.4). As τ_ahermal_ > τ, the time between the pulses is sufficiently long to carry the heat away from the focal volume before the arrival of the next pulse.

### Structural features of the FSL modified region using 100 kHz PRF

**Figure 4:**
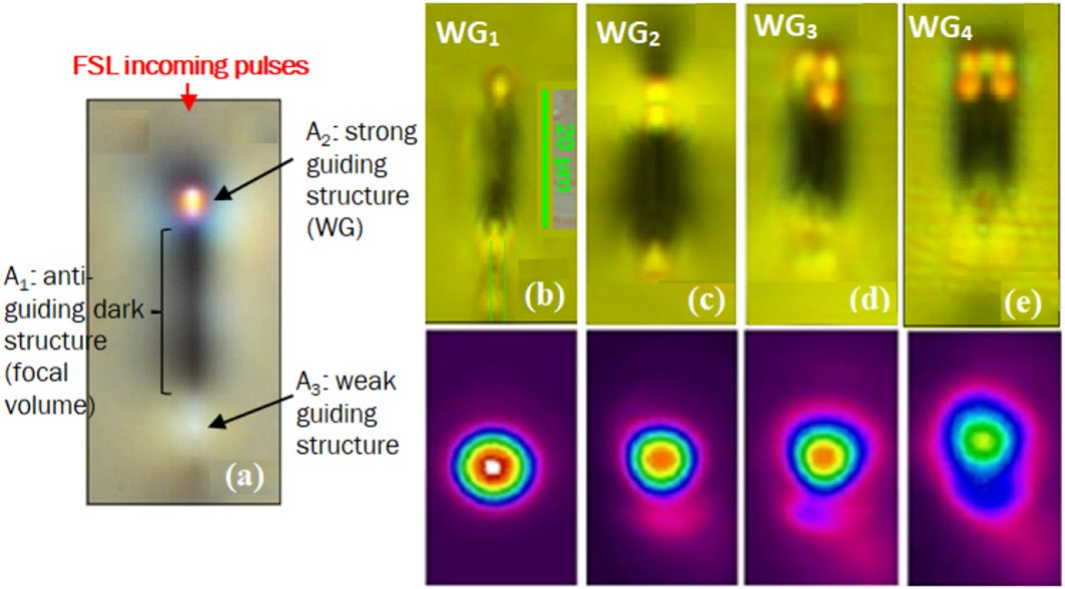
(**a**) 50 × bright field microscopy image illustrating the structural features of the single line FSL modified region (100 kHz PRF, 200 nJ pulse energy). The dark structure A_1_ is the focal volume. A_2_ above A_1_ is the WG due to its strong guiding capability. A_3_ below A_1_ is a weaker guiding structure. The red arrow indicates the direction of the FSL incoming pulses. (**b**–**e**) are 50 × brightfield microscopy images of the modified regions (cross-sectional view in top row). (**b**) single line (**c**) double line (2 single lines inscribed 5 μm apart). (**d**) triple line (3 single lines 5 μm apart) (**e**) 2 × 2 array of single lines written 5 μm apart. The 2nd row represents the corresponding 1550 nm beam profiles of the WGs in the strong guiding structures.

For initial index modifications within the bulk GPGN glass, intermediate pulse energies of 80 nJ and 100 nJ are selected. 100 nJ appeared to be the approximate threshold pulse energy for producing observable modified regions in the glass. To write more prominent regions in GPGN glass the writing pulse energy is increased to 200 nJ, i.e. twice the writing threshold. Figure 4a illustrates the structural features of the single line FSL modified region with 200 nJ pulse energies. The red arrow indicates the direction of the incoming FSL pulses. The modified region in the athermal regime is composed of three structures,

(i) An elongated non-guiding dark structure at the focal volume, labelled as A_1_ in Fig. 4a.
(ii) A bright guiding structure A_2_ above A_1_. A_2_ is referred to as WG due to its strong ability to guide light, and
(iii) A weak guiding structure A_3_ below A_1_.

Figure 4b–e represent the FSL modified regions inscribed with (b) single lines, (c) double lines (i.e. 2 single lines inscribed 5 μm apart) (d) triple lines (i.e. 3 single lines inscribed 5 μm apart), and (e) 2 × 2 array of single lines inscribed with 5 μm spacing. The bright guiding structures formed through these inscription approaches are referred to as WG_2_, WG_3_, and WG_4_, respectively.

As discussed earlier in results “Experimental methods”, the high peak power of FSLs generates a plasma in the focal volume which melts the glass. The melted glass immediately cools down as the sample moves away from the FSL beam path. A less dense glass volume leads to a lower refractive index and thus results in the formation of an anti-guiding or dark structure (A_1_ in Fig. 4a) at the FSL irradiated area^23,30^.

As the P_FSL_ (results “Experimental methods”) at 100 kHz PRF (~ 800 kW) is far above the estimated P_critical_ (~ 40 kW) for lead-germanate, the low self-focusing threshold is most likely the cause of elongation of A_1_ (Fig. 4a). Similar A_1_ structures (with varying elongation lengths depending on the strength of self-focusing) are also reported earlier in other germanate glasses and crystals such as in^12,15,31^.

A positive index change is observed at both ends of A_1_ (Fig. 4a). A_2_ near the surface of the sample (Fig. 4a) is found to strongly guide the light and is likely attributable to the lower density of A_1_^30^. As the pulse continues to propagate deeper, it is spatially degraded due to passing through the ionization that occurs at A_1_ and results in the formation of a diffused structure A_3_ (Fig. 4a).

WG_1_ in Fig. 4b inscribed using 200 nJ pulse energy (100 kHz) results in a small bright guiding structure closer to the surface with an apparent diameter of ~ 4 µm (Table 1). To inscribe WGs with increased beam diameters, multiple parallel lines are inscribed; double lines inscribed 5 μm apart (Fig. 4c), triple lines with 5 μm spacing (Fig. 4d), and 2 × 2 array of single lines with 5 μm spacing (Fig. 4e). As expected, the diameter of the guiding regions increases from 4 to 10 µm while remaining single mode. The maximum NA of the largest WG (i.e. WG_4_) written with the 100 kHz FSL (pulse energy = 200 nJ) is measured to be 0.028 with an estimated refractive index change (∆n) of ∆n ~ 1 × 10^–4^. For 100 kHz PRF, the maximum FSL pulse energy of 200 nJ is not sufficient to induce heat accumulation in GPGN glass. However, as observed for silicate glasses in^8,32^, increasing the pulse energy above 200 nJ can result in sufficient thermal diffusion to initiate weak to modest heat accumulation even at low PRF of 100 kHz (depending on the focusing conditions). This in turn can also lead to larger ∆n and WG dimensions and needs to be explored further for GPGN glass.

**Table 1:** PL and CL through 100 kHz, 200 nJ written WGs at 1550 nm wavelength. FL for the Yb^3+^:GPGN glass is (0.73 ± 0.05) dB. PL is the WG PL calculated from TL using PL = TL/d where d = 1.05 cm is the length of the WGs.

WG		WG diameter (μm)	CL ± 0.05 (dB)	PL (dB/cm)	Laser operation
WG_1_	Single line	4 µm × 4 µm	1.33	0.80 ± 0.07	No
WG_2_	Double lines (5 μm apart)	6 µm × 4 µm	1.01	0.91 ± 0.04	
WG_3_	Triple lines (5 μm apart)	8 µm × 6 µm	0.85	1.08 ± 0.06	
WG_4_	2 × 2 array of four single lines (5 μm apart)	10 µm × 9 µm	0.79	1.28 ± 0.07	

### PL in the athermally inscribed WGs and near-IR WG laser operation

The PL values of the inscribed 100 kHz PRF WGs are (are listed in Table 1). Inscribing multiple lines at 100 kHz PRF although increases the guiding cross-sections for WG_2_-WG_4_ compared to WG_1_ but is also accompanied by higher PL values (Table 1). The higher PL values are attributed to increased scattering that may arise due to the offset of 5 μm between the written lines being slightly greater than the single WG diameter (~ 4 μm).

No laser operation is observed in any of the 100 kHz written WGs due to the combination of PL in the cavity and high CL while launching into the small diameter WGs (e.g. CL + TL ~ 2.8 dB for single line induced WG).

### Thermal regime (1 MHz and 5 MHz)

In the thermal writing regime, the time between the FSL pulses reaching the focal volume is small compared to the diffusion time of the glass (i.e. ${\uptau }_{\mathrm{thermal}}\le$ 1 μs). This results in accumulation of more heat in the focal volume with each incoming FSL pulse. With higher cumulative heating in the focal volume (depending on the PRF and pulse energy) compared to the athermal regime, the electron density (plasma) in the FSL irradiated area also increases, heats up and eventually melts larger volume of the glass. When the sample moves away from the FSL beam path immediate quenching of the melted glass takes place which leads towards the formation of elliptical-shaped annulus structures in the vicinity of the focal volume with varying apparent index layers (i.e. dark and bright layers). The modified region in the thermal writing regime is therefore referred to as the heat accumulated region.

For 1 MHz PRF, the time between the incoming FSL pulses, τ_thermal (1 MHz)_ is 1 μs and is almost the same as the diffusion time of the GPGN glass (τ ~ 0.8 μs). Depending on the FSL pulse energy 1 MHz is an intermediate regime between thermal and athermal. In other words, the modified region at low pulse energies in 1 MHz PRF appears similar as in athermal regime. As the pulse energy increases the athermally modified region gradually transforms to the heat accumulation/ thermal region with varying index layers.

In contrast to 1 MHz, the modified region with 5 MHz is predominantly a heat accumulated region with complex structural features of bright and dark layers (discussed in next section). This is because the time between the 5 MHz PRF (τ_thermal (5 MHz)_ = 200 ns) is much less than the diffusion time of the glass (~ 0.8 μs). More heat starts accumulating in the focal volume and eventually produces larger volume of melted glass (compared to 1 MHz) which upon quenching results in larger heat accumulated regions.

Maximum NA of the largest WG in the thermal writing regime increases to 0.057 for WG_10_ compared to the athermally inscribed WGs. This gives an estimated refractive index contrast (∆n) of ~ 5 × 10^–4^ for thermally induced WGs.

### Structural features of FSL modified region in 1 MHz PRF

**Figure 5:**
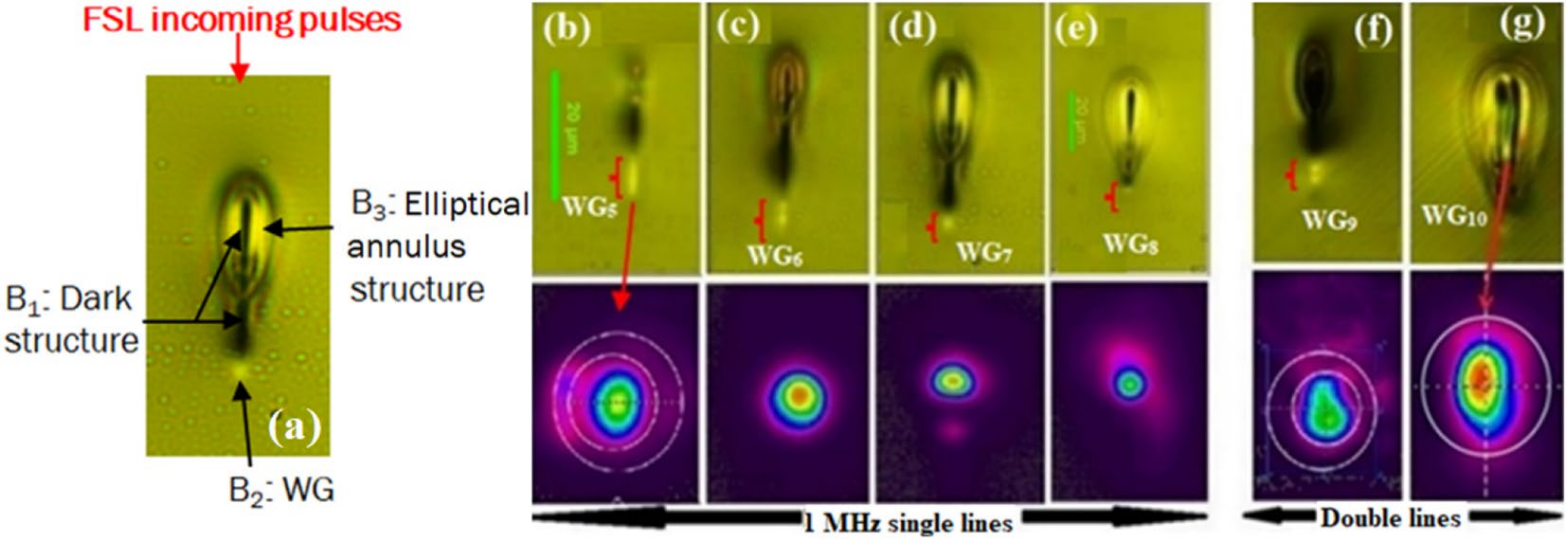
(**a**) 20 × Bright field microscopy image illustrating the structural features of the single line FSL modified region (1 MHz PRF, 150 nJ pulse energy). The dark structure B_1_ is the focal volume. B_2_ below B_1_ is the WG due to its strong guiding capability. B_3_ around the upper end of B_1_ is elliptical-shaped annulus structure which comprises of varying index layers. The red arrow indicates the direction of FSL incoming pulses. (**b**–**e**) 20 × Brightfield microscopy structural features (cross-section) of : (**b**–**e**) single line FSL modified regions (cross-sectional view in top row) with pulse energies varying from 50 to 200 nJ with 50 nJ intervals with the resulting WGs sequentially named as WG_5_-WG_8_, and (**f**–**g**) double line FSL modified regions with 100 nJ and 200 nJ pulse energies with guiding structures referred to as WG_0_ and WG_10_, respectively. The 2nd row represents the corresponding 1550 nm beam profiles of the WGs in the strong guiding structures below B_1_.

1 MHz single and double line FSL modified regions are inscribed in GPGN glass with pulse energies varying from 50 to 200 nJ with 50 nJ interval (Fig. 5a–g). The guiding structures (WGs marked in red) formulated in the single line and double line FSL modified regions are named as WG_5_-WG_10_ for pulse energies varying from 50 to 200 nJ. Figure 5a illustrates the 1 MHz PRF structural features induced by the single line FSL modified region with 150 nJ pulse energy. A red arrow indicates the direction of incoming FSL pulses. The modified region is composed of the following three structures:

(i) An elongated dark structure (lower refractive index) at the focal volume in the middle of the modified region, labeled as B_1_ in Fig. 5a. Even at a low pulse energy of 50 nJ, the peak laser power (P_FSL_ ~ 200 kW) exceeds the self-focusing threshold limit and results in the elongation of B_1_ (described in “Experimental methods”).
(ii) A bright guiding structure (WG) labeled as B_2_ below B_1_.
(iii) An elliptical-shaped annulus structure B_3_ with varying index layers above B_1_. The heat accumulation in B_1_ results in the formation of B_3_.

The 1 MHz PRF, 50 nJ single line FSL modified region (Fig. 5b) is closer in form to the 100 kHz modified region with B_2_ as WG_5_. As the pulse energy increases from 50 to 100 nJ (Fig. 5c) more heat accumulates in B_1_ and the characteristic heat accumulated structural features (B_3_ in Fig. 5a) around B_1_ start appearing along with the positive index guiding structure (WG_6_) below B_1_. As the pulse energy further increases (Fig. 5c–e), more heat is deposited in B_1_ resulting in the formation of larger and brighter B_3_. In addition to increasing B_3_, B_2_ below B_1_ gradually decreases in size as the pulse energy increases (as is evidenced from the brightfield microscopic images and 1550 nm beam profile in Fig. 5b–e. In short, smooth transition from thermal diffusion at low pulse energies to heat accumulation at higher pulse energies is observed in 1 MHz PRF modified regions thus reflecting 1 MHz to be an intermediate regime. For 1 MHz PRF, 100 nJ is observed to be the threshold pulse energy to initiate heat accumulation in GPGN glass (Fig. 5b–c). Similar transition from thermal diffusion (athermal regime) to heat accumulation (thermal regime) is previously studied in silicate glasses and fused silica from 0.1 to 5 MHz^8,24,32^. For silicate glasses the transition threshold is observed for PRF ~ 0.2 MHz while for fused silica the transition threshold PRF is ~ 0.5 MHz under the same focusing conditions.

To increase the WG diameter in the 1 MHz PRF we also inscribed double line FSL modified regions in GPGN glass with 5 µm spacing (Fig. 5f,g). The resulting guiding structures are referred to as WG_9_ and WG_10_ in Fig. 5f,g and are of larger diameters compared to single line induced WGs in Fig. 5a–e. The 1550 nm beam profiles for 1 MHz written structures reflect that the inscribed WGs are single mode in nature.

### PL in 1 MHz induced WGs and near-IR WG laser operation

**Figure 6:**
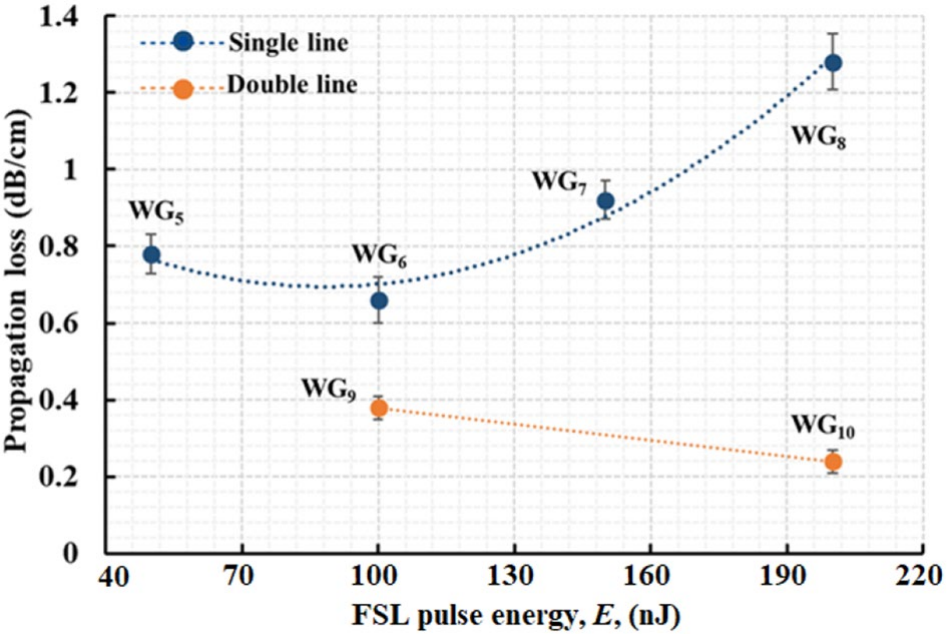
PL in single line induced WGs (WG_5_–WG_8_ in blue) and double line induced WGs (WG_9_–WG_10_ in brown) as a function of FSL pulse energy. Minimum PL of ~ 0.2 dB/cm is measured for WG_10_.

The measured PL and the laser slope efficiencies for the 1 MHz PRF induced WGs are listed in Table 2. Figure 6 plots the PL for the 1 MHz inscribed WGs. The data in blue (Fig. 6) is the PL for single line induced WGs using 1 MHz PRF with varying FSL pulse energies. The brown plotted data is the PL for WGs induced by writing double line regions. As shown in Table 2, PL in WG_5_ is greater than WG_6_. For WG_7_ and WG_8_ the PL starts increasing again as the guiding structure deeper into the sample decreases (in size) giving way to the formation of heat accumulated region which becomes more prominent (with no defined guiding structure) as the pulse energy increases (Fig. 5b–e).

**Table 2:** CL, PL and laser slope efficiencies in 1 MHz written WGs in Yb^3+^: GPGN glass at 1550 nm. FL for the Yb^3+^:GPGN glass is (0.73 ± 0.05) dB. PL is the WG PL calculated from TL using PL = TL/d where d = 1.05 cm is the length of the WGs.

WG	Pulse Energy (nJ)	CL ± 0.05 (dB)	PL (dB/cm)	Laser slope efficiency (%η)
WG_5_	50	0.92	0.74 ± 0.05	7
WG_6_	100	0.68	0.63 ± 0.06	10
WG_7_	150	0.95	0.88 ± 0.05	No lasing
WG_8_	200	1.24	1.22 ± 0.07	No lasing
WG_9_	100	0.85	0.34 ± 0.03	19
WG_10_	200	0.63	0.22 ± 0.03	28

WG_9_ and WG_10_ are written with the same pulse energies as WG_6_ and WG_8_, respectively, however, the PL measured for the double line WGs are observed to be relatively lower. This is attributed to the formation of larger and smoother WGs (∆n ~ 5.4 × 10^–4^ for WG_10_). A low PL of ~ 0.2 dB/cm is measured for WG_10_ which is the lowest PL observed in GPGN glass to date.

**Figure 7:**
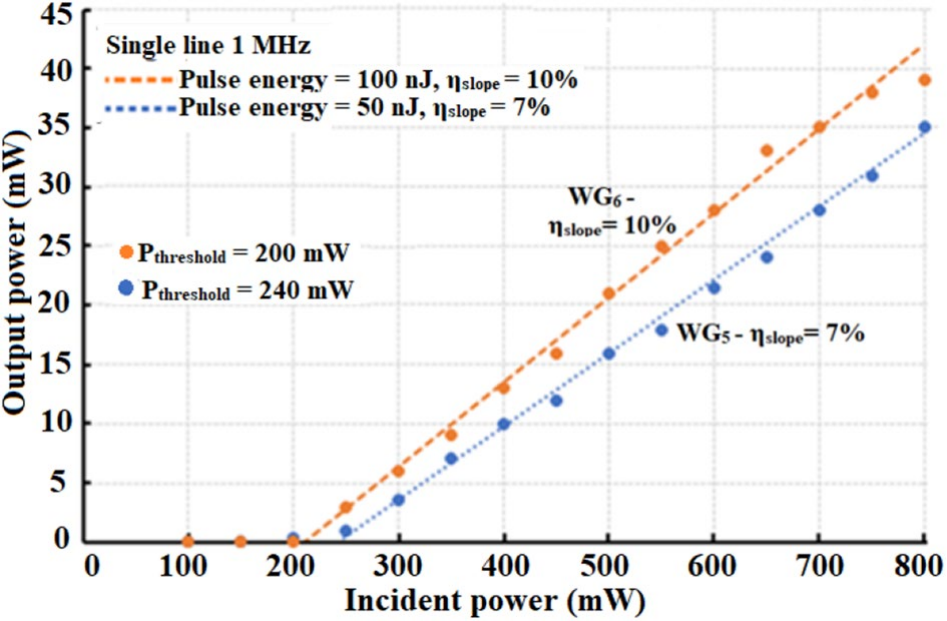
~ 1 µm laser operation in WG_5_ and WG_6_ with slope efficiencies, η_slope_, 7% and 10%, respectively.

**Figure 8:**
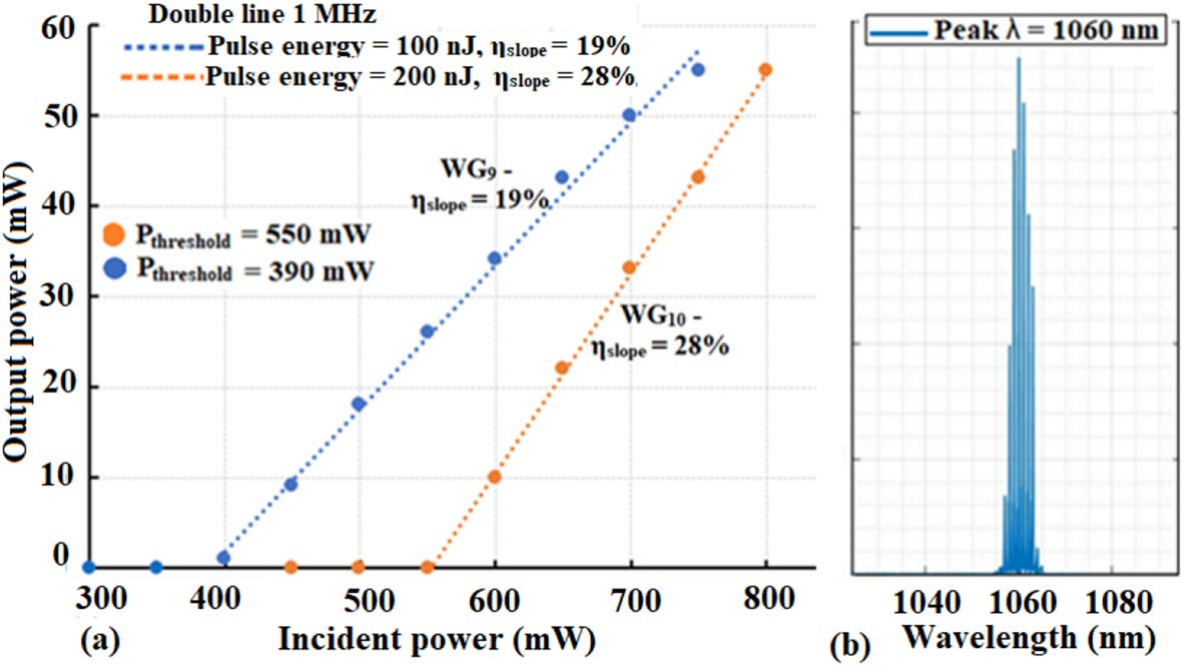
(**a**) Laser operation in double line induced WG_9_ and WG_10_ with improved 19% and 28% slope efficiencies. At P_in_ > 800 mW, the laser signal power dropped due to thermal lensing within the WGs. (**b**) WG_10_ laser operation at 1060 nm with a FWHM ~ 5 nm.

Laser operation at ~ 1 µm (using the setup in Fig. 3) is achieved in WG_5_, WG_6_, WG_9_ and WG_10_ with ~ 7%, 10%, 19% and 28% slope efficiencies, respectively (Figs. 7 and 8). WG_7_ and WG_8_ could not be operated as a laser due to higher PL. The broad spectral output from WG_10_ is shown in Fig. 8b and is centered at ~ 1060 nm with a full width half maximum value of ~ 5 nm.

### Structural features of FSL modified region in 5 MHz PRF

**Figure 9:**
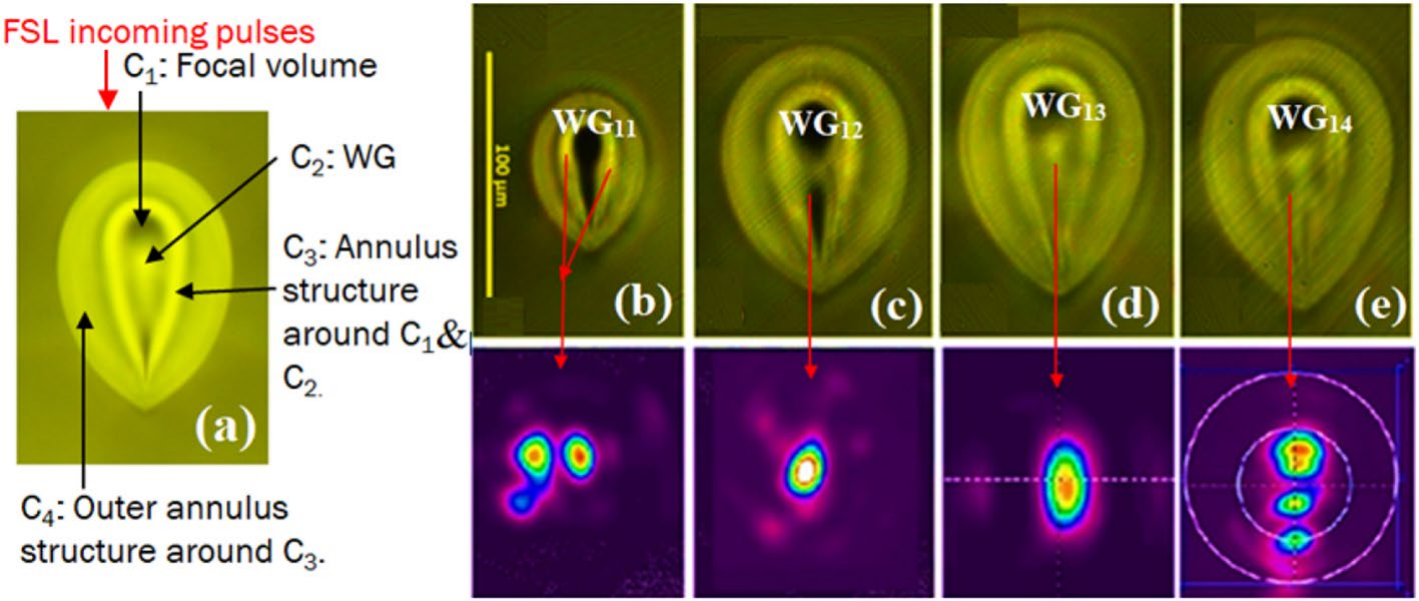
(**a**) 20 × Bright field microscopy image (cross-sectional view) of single line FSL modified region in 5 MHz PRF, 100 nJ pulse energy. The image illustrates the different structures of the heat accumulated region: the dark structure is the focal volume C_1_, C_2_ is the bright guiding structure (WG) originating from the center of C_1_ at higher pulse energies. C_3_ is annulus structure with varying index layers around C_1_ and C_2_. C_4_ is the outer annulus structure, although bright, it is not suitable for laser applications. (**b**–**e**) 20 × Brightfield microscopy image of the 5 MHz FSL single line FSL modified regions with pulse energies ranging from 60 to 120 nJ with 20 nJ intervals. The bright structures in C_1_ (red arrows) are the guiding structures named WG_11_–WG_14_. The 2nd row illustrates the 1550 nm beam profiles in the inscribed WGs.

5 MHz PRF single line FSL modified regions are inscribed in GPGN glass with pulse energies varying from 60 to 120 nJ with 20 nJ intervals (Fig. 9a–e). The guiding structures (WG) formulated in the single line FSL modified regions are represented as WG_11_-WG_14_ (red arrows in Fig. 9b–e for pulse energies varying from 60 to 120 nJ, respectively. Figure 9a illustrates the structural features of the single line FSL modified region. A red arrow indicates the direction of incoming FSL pulses. The modified region is a heat accumulated region with varying index layers. The heat accumulated region comprises the following four structures located radially around the central focal volume (Fig. 9a):

(i) An elongated dark structure at the focal volume in the center of the modified region labeled as C_1_ in Fig. 9a. As explained earlier, the dark structure represents the low refractive index at the FSL exposed area. The elongation of the focal volume is the result of strong self-focusing even at low pulse energy of 60 nJ.
(ii) A bright circular-shaped guiding structure C_2_.
(iii) An elliptical shaped annulus structure C_3_ around C_2_.
(iv) An outer annulus structure C_4_ around C_3_.

The 5 MHz modified region inscribed with 60 nJ pulse energy (Fig. 9b) is similar to the 1 MHz PRF single line FSL modified region written with 200 nJ pulse energy (Fig. 5e), but with larger dimensions and a more dense elliptical-shaped annulus structure around C_1_. The additional heat accumulation in C_1_ compared to B_1_ [as τ_thermal(5 MHz)_ (200 ns) ≪ τ_thermal (1 MHz)_ (1 μs)] results in much higher plasma density which upon immediate quenching of the glass melt densifies around C_1_ and results in C_3_. The uniform spherical cooling of the focal volume leads to the formation of non-uniform bright and dark index layers surrounding C_1_ (Fig. 9b–e).

The 1550 nm beam profile through the 60 nJ inscribed WG_11_ is observed to be multimode due to light launching into the C_3_ structure (Fig. 9b). With the increase in the FSL pulse energy from 60 to 80 nJ a single mode C_2_ structure (~ 12 μm) originates from the center of the modified region (Fig. 9c). As the pulse energy further increases, C_2_ enlarges in size such that it becomes multimode again (Fig. 9d–e).

### PL in 5 MHz induced WGs and near-IR WG laser operation

**Figure 10:**
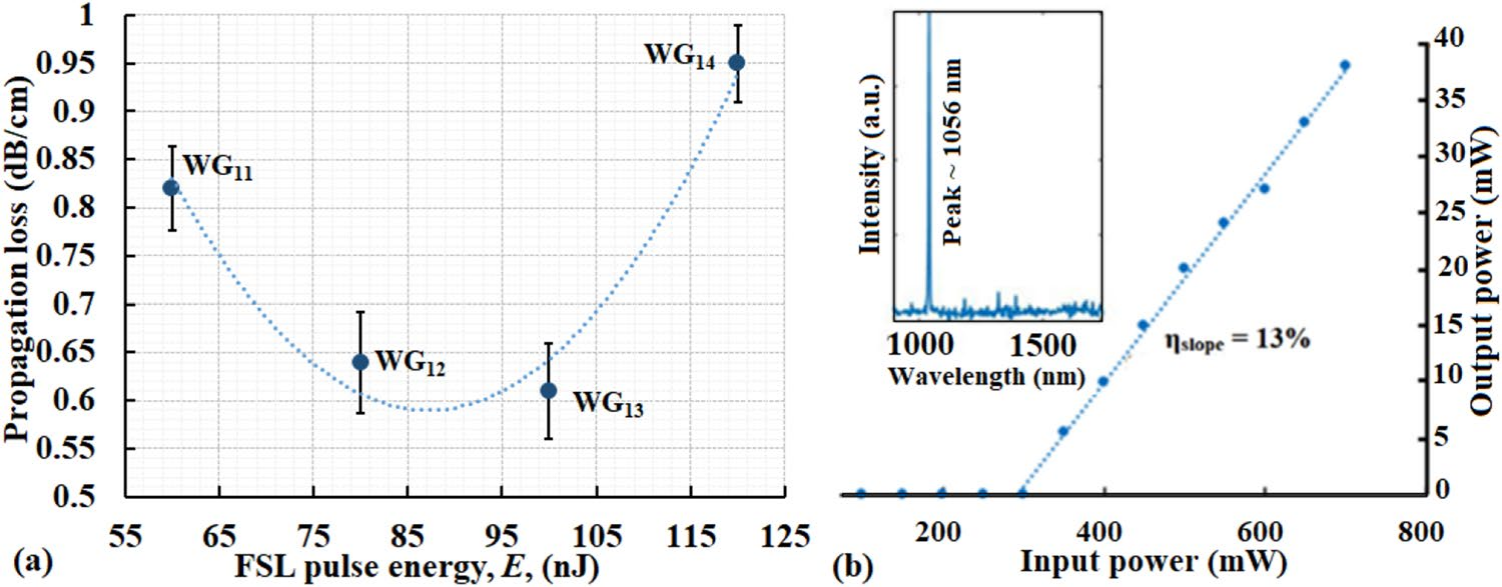
(**a**) PL in the 5 MHz inscribed WGs as a function of the FSL pule energy. (**b**) 1056 nm laser operation in WG_12_ with max. η_slope_ = 13% slope efficiency and P_threshold_ ~ 250 mW.

Table 3 lists the PL and laser slope efficiencies measured in the 5 MHz inscribed WGs. The PL reduces from WG_11_ to WG_13_ as the FSL pulse energy increases from 60 to 100 nJ (Fig. 10a). WG_11_ which is multimodal at 1550 nm has a higher PL compared to WG_12_. With the formation of a smooth C_2_ structure for WG_12_ the PL is reduced until a lowest loss of 0.58 dB/cm is obtained for WG_13_ (pulse energy = 100 nJ). With further increase in FSL pulse energy to 120 nJ, C_2_ represented as WG_14_ again turns multimode and thus PL increases again (Fig. 10a).

**Table 3:** Measured CL, PL and laser slope efficiency in the fundamental mode of the 5 MHz written WGs in Yb^3+^: GPGN glass at 1550 nm. FL for the Yb^3+^:GPGN glass is (0.73 ± 0.05) dB. PL is the WG PL calculated from TL using PL = TL/d where d = 1.05 cm is the length of the WGs.

WG	Pulse energy (nJ)	CL ± 0.05 (dB)	PL ± 0.05 (dB/cm)	Laser slope efficiency (% η)
WG_11_	60	1.12	0.72	9
WG_12_	80	0.98	0.61	12
WG_13_	100	1.29	0.58	13
WG_14_	120	1.08	0.97	4

All the WGs are found to lase at the expected wavelength of ~ 1 µm with slight variation in operating wavelengths depending upon the WG diameter (i.e. due to the population inversion ratio modifying the ground state absorption). The highest laser slope efficiency of ~ 13% is achieved for WG_13_ with the lowest PL and operating at a wavelength of 1056 nm (Fig. 10b).

## Conclusion

We fabricated a range of waveguides in a RE-doped lead-germanate GPGN glass using FSL in three different PRF i.e. 100 kHz (athermal regime), 1 MHz and 5 MHz (thermal regime), and verified their lasing capability in a near-IR region. The aim of the study is to optimize the FSL parameters in such a way that low propagation loss guiding structures are induced in GPGN glass for their efficient utilization in near to mid-IR laser applications. Irradiating GPGN glass with FSL pulses results in positive refractive index change in the GPGN glass in the vicinity of focal volume (above, below or around the focal volume depending on the writing regime). The refractive index contrast (∆n) for athermal waveguides is ~ 1 × 10^–4^ while for thermal waveguides ∆n increases to ~ 5 × 10^–4^. It is concluded that the heat accumulation in thermal regime is responsible for the generation of smooth guiding structures with large ∆n. This in turn suppresses the impact of aberrations and self-focusing on the guiding region thus reducing the PL in thermally inscribed WGs in the GPGN glass. The lowest loss of ~ 0.2 dB/cm at 1550 nm is measured in a 1 MHz written double-line WG. To the best of our knowledge, the PL of 0.2 dB/cm is the lowest loss reported in a FSL inscribed WG in germanate glass and is equivalent to the PL observed in a silica glass^33^. Near-IR waveguide laser operation (~ 1060 nm) in the lowest loss 1 MHz written WG results in a best achieved laser slope efficiency of ~ 28% which is the highest yet reported in GPGN glass. Further improvement in slope efficiency of the Yb^3+^ doped GPGN glass waveguide laser can be realized by lowering the propagation losses by balancing the FSL parameters in such a way that self-focusing is minimized and symmetrical waveguide structures are inscribed.

There is still room for further improvement in the WG laser slope efficiencies by reducing the PL in WGs with careful tuning of variable FSL parameters in such a way that induced WGs are still single mode. Moreover, the self-focusing, which arises due to the interaction of high power FSL pulses with GPGN glass, needs to be further explored (theoretically and experimentally) to minimize its impact on the inscribed WGs in GPGN glass (more specifically in athermal regime).

## Supplementary Information

Supplementary Information.
