# Author Correction: Femtosecond laser induced low propagation loss waveguides in a lead-germanate glass for efficient lasing in near to mid-IR

**DOI:** 10.1038/s41598-023-37789-4

**Published:** 2023-06-30

**Authors:** Mamoona Khalid, George Y. Chen, Heike Ebendorff‑Heidepreim, David G. Lancaster

**Affiliations:** 1 Laser Physics and Photonics Devices Laboratory (LPPDL), University of South Australia, Mawson Lakes, SA 5095 Australia; 2 Institute for Photonics and Advanced Sensing and School of Physical Sciences, University of Adelaide, Adelaide, SA 5000 Australia

Correction to: *Scientific Reports* 10.1038/s41598-021-90249-9, published online 24 May 2021

The original version of this Article contained an error in the legend of Figure 1a, where a reference was omitted. The reference is listed below as Reference 20.

20. Grenier, J.R., Fernandes, L.A. & Herman, P.R. Femtosecond laser writing of optical edge filters in fused silica optical waveguides. *Opt. Express* **21**, 4493–4502. https://doi.org/10.1364/OE.21.004493 (2013).

“(**a**) FSL material processing setup for WG writing. The sample is moved transversely with respect to the FSL focus to inscribe tracks/lines of index modifications. The cross-section of the single line FSL modified region presented in the sample is the heat accumulated region (thermal writing regime). (**b**) Ray schematic showing the estimated change in writing depth [from d = 150 μm to d’ = 234 μm (FSL estimated focus)] through a low refractive index n = 1.51 index matching oil to a high refractive index sample (n’ = 1.82). θ and θ’ are the angle of refraction of the focusing beams (for actually set and estimated writing depths) with respect to incidence normal.”

now reads:

“(**a**) FSL material processing setup for WG writing. The sample is moved transversely with respect to the FSL focus to inscribe tracks/lines of index modifications. The cross-section of the single line FSL modified region presented in the sample is the heat accumulated region (thermal writing regime). Reproduced with permission from^20^. (**b**) Ray schematic showing the estimated change in writing depth [from d = 150 μm to d’ = 234 μm (FSL estimated focus)] through a low refractive index n = 1.51 index matching oil to a high refractive index sample (n’ = 1.82). θ and θ’ are the angle of refraction of the focusing beams (for actually set and estimated writing depths) with respect to incidence normal.”

As a result, the subsequent References have been renumbered.

The original Article has been corrected.

