# Supplementary material for: Femtosecond laser induced low propagation loss waveguides in a lead-germanate glass for efficient lasing in near to mid-IR
Source: Sci Rep. 2021 May 24;11:10742. doi: 10.1038/s41598-021-90249-9 (PMC8144420; doi:10.1038/s41598-021-90249-9)
Supplement: Supplementary file 1 — Supplementary Information. [file 41598_2021_90249_MOESM1_ESM.docx]

**Supplement 1:** Femtosecond laser induced low propagation loss waveguides in a lead-germanate glass for efficient lasing in the mid-IR

**Section 2: Waveguide characterization.**

**Propagation loss (PL) measurements through the WGs**

Figure 2 in the main manuscript gives the total probe beam loss (L) characterization setup utilized for the measurement of CL and PL in the waveguides (WGs). The following procedure is followed to compute L contributions in the forward (‘a’ to ‘e’) and backward (‘e’ to ‘b’) beam paths as identified in section 2 of the main manuscript in WG characterization section. It is worth mentioning here that all the components are not placed in the setup at the same time but the following sequence of placing components and recording power measurements at each point a, b, c, d, and e is followed to calculate different contributions of L in the setup of Fig. 2.

1. 1550 nm probe beam power (P_c_) at point ‘c’ is measured using a power meter. PBS and λ/4 waveplate are then inserted in the setup and a 97% reflecting mirror (R) @ 1550 nm (M_1_) is placed at point ‘c’ to retroreflect the 1550 nm beam to the point ‘b’ (corresponding power, P_b_). This step estimates OL (dB) through PBS and waveplate using Eq. (S1). ML is the 3% mirror loss.

$\text{L}_{1}\text{= 10log}_{10}\left(\frac{\text{P}_{\text{b}}}{\text{P}_{\text{c}}}\right)\text{ = OL + ML}$ (S1)

1. M_1_ from point ‘c’ is then removed and P_c_ is measured with the power meter. The WG sample is then introduced after point ‘c’ (without the focusing lens so that c = d) and aligned (w.r.t He-Ne laser) such that the 1550 nm beam passes through the bulk glass and received at point ‘e’. For this step P_c_ = P_d_ in Fig. 2 is considered as the input to the bulk glass and L_2_ at ‘e’ (with respect to ‘c’) is measured using Eq. (S2). FL is then computed considering TL ~ 0 using Eq. (S2) (TL in the high purity bulk Yb^3+^:GPGN with smooth surface finishing is measured as 0.02 dB which is lower than the detection limit of this method (i.e. 0.05 dB), we therefore consider TL ~ 0)

$\text{L}_{2}\text{= 10log}_{10}\left(\frac{\text{P}_{\text{e}}}{\text{P}_{\text{c}}}\right)\text{ = FL}$ (S2)

1. 1550 nm probe beam is then launched into the WGs by introducing a focusing lens after point ‘c’ (lens is ‘C’ coated so the loss is negligible). M_1_ = M_2_ is removed from the setup. The intensity of the probe beam at point ‘d’ and ‘e’ (i.e. P_d_  after the focusing lens is the input and P_e_ at the end of WG is the output) is measured using a phosphor-coated CCD camera beam profiler (SP503U) to detect only the fundamental mode of the waveguides by adjusting the built-in aperture of the beam profiler. CL was introduced in the forward L_3_ at point ‘e’ due to the poor launch conditions into the WGs. FL was already known from the previous step 3.

$\text{L}_{3}\text{= 10log}_{10}\left(\frac{\text{P}_{\text{e}}}{\text{P}_{\text{d}}}\right)\text{ = CL + TL + FL}$ (S3)

1. M_2_ is then placed at point ‘e’ (just after the waveguide end face) in such a way that the probe beam travels back through the WG, lens, λ/4 waveplate and PBS to the point ‘b’. For returning beam towards point ‘b’ the focusing lens works as a collimating lens and CL is considered negligible. The loss at point ‘b’ with respect to input point ‘d’ is measured using Eq. (S4).

$\text{L}_{4}\text{= 10log}_{10}\left(\frac{\text{P}_{\text{b}}}{\text{P}_{\text{d}}}\right)\text{ = 2TL + 2FL + CL + OL + ML}$ (S4)

Utilizing Eq. S1 to Eq. S4, TL in the WG is measured as:

$\text{TL (dB) = L}_{4}-\text{ L}_{3}-\text{ L}_{2}-\text{ L}_{1}$ (S5)

From TL in Eq. (S5), PL is extracted by dividing TL with the length of the WG (d) in cm, i.e. PL (dB/cm) = TL/d. CL is finally measured using Eq. (S6)

$\text{CL}\left(\text{dB}\right)\text{ = 2L}_{3}\text{+ L}_{1}\text{- L}_{4}$ (S6)
